# Web-Based Patient-Reported Outcome Measures for Personalized Treatment and Care (PROMPT-Care): Multicenter Pragmatic Nonrandomized Trial

**DOI:** 10.2196/19685

**Published:** 2020-10-29

**Authors:** Afaf Girgis, Ivana Durcinoska, Anthony Arnold, Joseph Descallar, Nasreen Kaadan, Eng-Siew Koh, Andrew Miller, Weng Ng, Martin Carolan, Stephen A Della-Fiorentina, Sandra Avery, Geoff P Delaney

**Affiliations:** 1 Centre for Oncology Education and Research Translation Ingham Institute for Applied Medical Research Sydney Australia; 2 Faculty of Medicine The University of New South Wales Sydney Australia; 3 Illawarra Cancer Care Centre, Wollongong Hospital Wollongong Australia; 4 Liverpool Cancer Therapy Centre, Liverpool Hospital Sydney Australia; 5 Centre for Oncology Informatics University of Wollongong Wollongong Australia; 6 School of Medicine Western Sydney University Sydney Australia; 7 Macarthur Cancer Therapy Centre, Campbelltown Hospital Sydney Australia

**Keywords:** patient-reported outcomes (PROs), eHealth, patient-centered care, electronic health record, nonrandomized controlled trial, emergency department presentations, pragmatic trial, symptom screening

## Abstract

**Background:**

Despite the acceptability and efficacy of e–patient-reported outcome (ePRO) systems, implementation in routine clinical care remains challenging.

**Objective:**

This pragmatic trial implemented the PROMPT-Care (Patient Reported Outcome Measures for Personalized Treatment and Care) web-based system into existing clinical workflows and evaluated its effectiveness among a diverse population of patients with cancer.

**Methods:**

Adult patients with solid tumors receiving active treatment or follow-up care in four cancer centers were enrolled. The PROMPT-Care intervention supported patient management through (1) monthly off-site electronic PRO physical symptom and psychosocial well-being assessments, (2) automated electronic clinical alerts notifying the care team of unresolved clinical issues following two consecutive assessments, and (3) tailored online patient self-management resources. Propensity score matching was used to match controls with intervention patients in a 4:1 ratio for patient age, sex, and treatment status. The primary outcome was a reduction in emergency department presentations. Secondary outcomes were time spent on chemotherapy and the number of allied health service referrals.

**Results:**

From April 2016 to October 2018, 328 patients from four public hospitals received the intervention. Matched controls (n=1312) comprised the general population of patients with cancer, seen at the participating hospitals during the study period. Emergency department visits were significantly reduced by 33% (*P*=.02) among patients receiving the intervention compared with patients in the matched controls. No significant associations were found in allied health referrals or time to end of chemotherapy. At baseline, the most common patient reported outcomes (above-threshold) were fatigue (39%), tiredness (38.4%), worry (32.9%), general wellbeing (32.9%), and sleep (24.1%), aligning with the most frequently accessed self-management domain pages of physical well-being (36%) and emotional well-being (23%). The majority of clinical feedback reports were reviewed by nursing staff (729/893, 82%), largely in response to the automated clinical alerts (n=877).

**Conclusions:**

Algorithm-supported web-based systems utilizing patient reported outcomes in clinical practice reduced emergency department presentations among a diverse population of patients with cancer. This study also highlighted the importance of (1) automated triggers for reviewing above-threshold results in patient reports, rather than passive manual review of patient records; (2) the instrumental role nurses play in managing alerts; and (3) providing patients with resources to support guided self-management, where appropriate. Together, these factors will inform the integration of web-based PRO systems into future models of routine cancer care.

**Trial Registration:**

Australian New Zealand Clinical Trials Registry ACTRN12616000615482; https://www.anzctr.org.au/Trial/Registration/TrialReview.aspx?id=370633

**International Registered Report Identifier (IRRID):**

RR2-10.1186/s12885-018-4729-3

## Introduction

Organizations delivering health care services increasingly incorporate patient-reported outcomes (PRO) to inform person-centered care and evaluate services. Well-implemented ePRO systems are associated with improved patient-provider communication, patient satisfaction [1], health-related quality of life [2,3], compliance with chemotherapy [3], earlier detection of relapse in patients with lung cancer [4], reduced emergency department (ED) presentations [5,6], and improved cancer survival [6,7]. However, implementation and effectiveness evaluations in real-world clinical practice settings are not well studied.

A systematic review of 6 reviews identified facilitators and barriers for the implementation of ePROs in health services, with 2 early stages in the implementation process being critical for organizational time and resource investment [8]. First, designing the processes for using PROs within an organization, with a focus on decisions about data use for clinical purposes, rather than just which PROs to collect and how to collect them. Second, preparing organizations and staff for using PROs, including highlighting their validity and value, training clinicians using them, and developing electronic systems that fit into the centers’ patient workflow.

Our team developed PROMPT-Care (Patient Reported Outcome Measures for Personalized Treatment and Care), an ePRO system for routinely collecting PROs remotely from home for patients with cancer. PROMPT-Care provides real-time feedback of results to the cancer care teams to inform patient-centered care and deliver evidence-based self-management information to address patient-reported problems [9]. PROMPT-Care is fully integrated into the electronic oncology information system (OIS), which is acceptable to patients and oncology staff and feasible to implement clinically [10].

This multicenter, pragmatic [11] nonrandomized intervention study aimed to successfully implement PROMPT-Care Version 2.0 [12] into existing clinical workflows, evaluate its effectiveness in a diverse population of patients with cancer, and explore the system utility from patient and health care professional perspectives. We hypothesized that PROMPT-Care intervention patients would have significantly fewer ED presentations during the study period compared to a usual care control group. The intervention was also expected to impact the time spent on chemotherapy and the number of health service referrals.

## Methods

### Study Design and Participants

The trial protocol details the study design [12]. Briefly, we conducted a pragmatic, nonrandomized trial among patients with cancer throughout all stages of their cancer care trajectory, with different tumor types and receiving active treatment or follow-up care between April 2016 and October 2018 at four public hospitals in New South Wales, Australia. This study design [11] was chosen to inform external validity and determine this care model’s suitability for broad cancer populations, including those in follow-up. Study participants were compared to the general population of patients with cancer receiving usual care at participating hospitals during the study period.

Eligible patients were adults with a confirmed diagnosis of a solid tumor and cognitively able to provide informed consent and complete the assessments in English. Patients without internet access outside the hospital were excluded. Patients with upcoming clinical appointments (treatment or follow-up) at participating hospitals were prescreened for eligibility by treating clinicians using lists extracted from the OIS every month and invited to participate by the nursing or research staff. Patients received written and verbal information about the study, provided their consent, and participated for a minimum of 6 months. Participants received monthly emails prompting them to complete the upcoming online assessment, with one reminder email sent a week later. The Human Research Ethics Committee of South Western Sydney and Illawarra Shoalhaven Local Health Districts (Reference No. HREC/15/LPOOL/287) granted ethics approval, and the trial was registered in the Australian New Zealand Clinical Trials Registry (ACTRN12616000615482).

### Intervention

**Figure 1:**
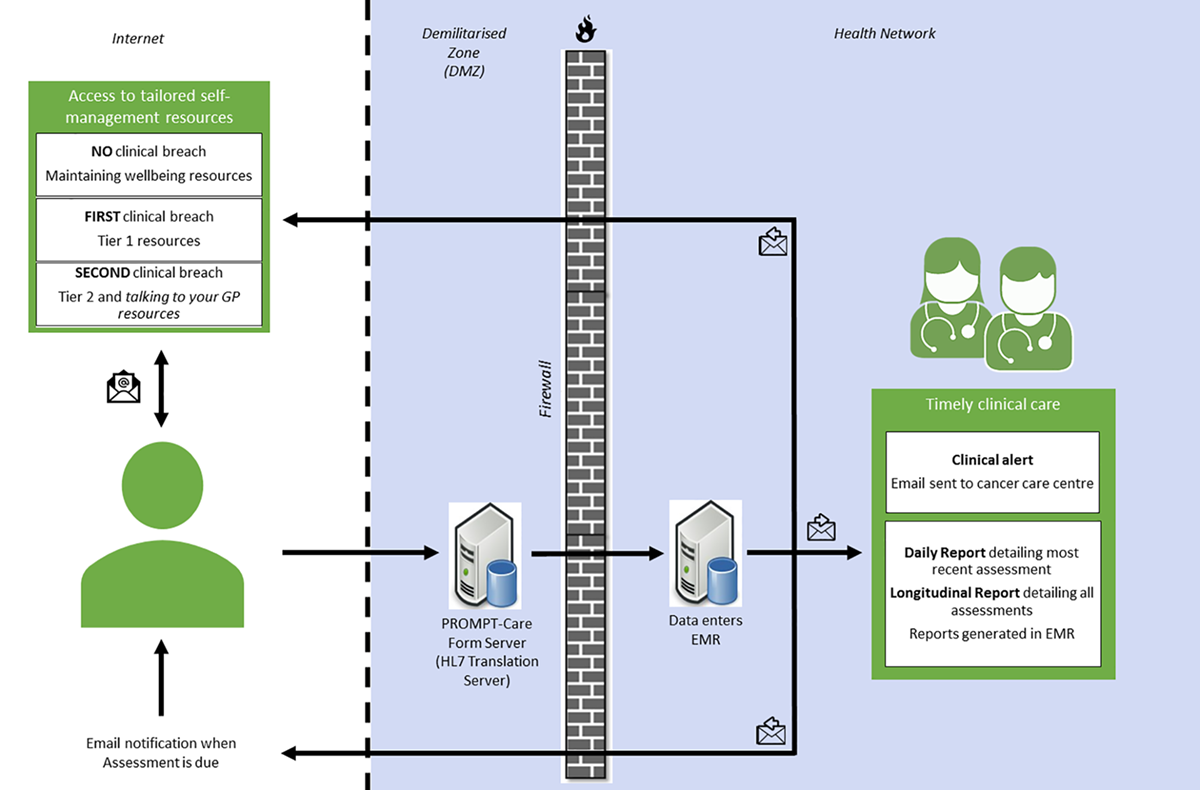
PROMPT-Care 2.0 system overview.

Four key intervention components were standardized across participating hospitals: ePROs, clinical feedback reports, clinical alerts, and patient self-management (Figure 1).

#### ePROs

Approximately once a month, patients were prompted via email to complete an online assessment of their physical and psychosocial well-being from home, which included the Distress Thermometer and checklist [13], the Edmonton Symptom Assessment Scale (ESAS) [14], and the Supportive Care Needs Survey-Screening Tool 9 [15]. Patients used an electronic device (eg, tablet, computer), with real-time electronic data transfer (using PROsaiq, DidymoDesigns) into the point-of-care OIS (MOSAIQ, Elekta Medical Systems), via automatic conversion into an Health Level Seven (HL7) message [16].

Patient privacy was maintained by ensuring the email did not contain identifiable patient information, in the event the email was intercepted or sent to the wrong email address. PROsaiq [16], which has restricted access, was located on a PROMPT-Care form server within the demilitarized zone of the health network. To complete an assessment, the patient opened the URL provided in the email, and the browser established a secure session to the PROMPT-Care system, where an SSL Certificate was installed. The patient was required to enter a unique medical record number and their surname to access the system. The two identifiers were chosen to match the survey results to the correct patient in the OIS. Following the assessment completion, data were translated into a HL7 message by the OIS HL7 translation server, which also sat behind the health network firewall. If the medical record number and surname attached to the HL7 message matched to a patient in the OIS, the survey results were loaded into the OIS under the patient record. If a mismatch occurred, the data were not loaded and a failure was recorded in a log file. No patient information was stored on the PROMPT-Care form server.

#### Clinical Feedback Reports

**Figure 2:**
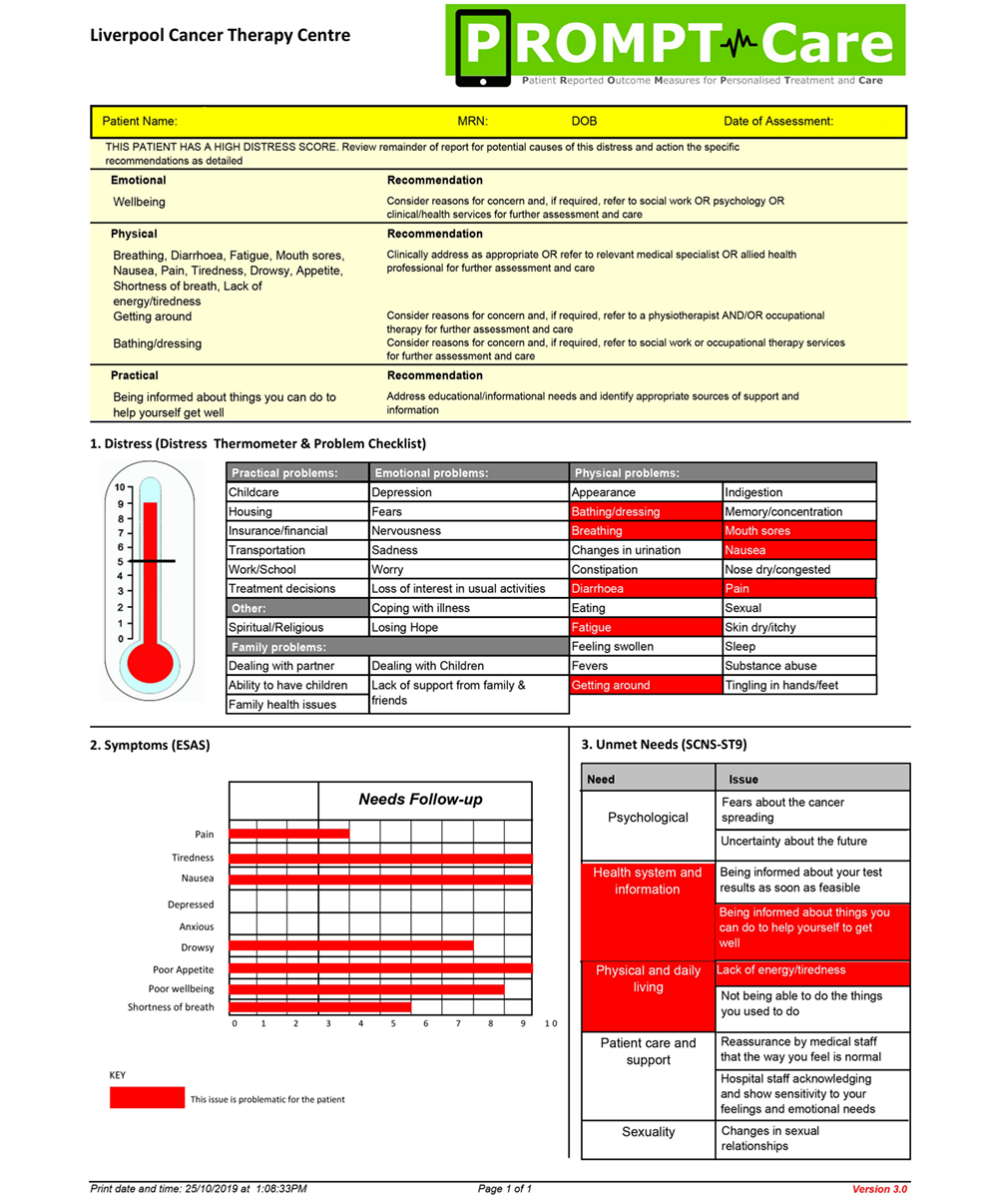
Sample clinical feedback report.

Any care team member could review the ePRO reports in real time with patients, or as required. The ePRO reports included (1) a 1-page summary of the most recent assessment results, including recommended clinical actions and referrals generated from algorithms to facilitate standardized care (Figure 2) [17,18], and (2) a longitudinal report of all assessments to date. Staff were oriented on clinical feedback report use and access at the start of the study, with a periodic refresher training and emails with brief instructions on accessing and using reports in practice throughout the trial.

#### Clinical Alerts

An email alert was automatically generated whenever any individual ePRO item score breached a predefined threshold on two consecutive assessments. This email was generated by a SMTP server within the health network, where only approved health email addresses were able to receive this email alert ensuring that patient identifying data were unable to leave the organization. A designated member of the care team reviewed emails at least once a day (Monday-Friday) and followed their cancer center’s standard clinical care pathway.

#### Patient Self-Management

Approved resources were hosted on a website to support patient self-management, [12,17], with domain-specific webpages—practical problems and emotional, physical, social and family well-being, and a “maintaining well-being” page to support general health. Immediately following ePRO completion, patients received an email with links to pages related to the above-threshold ePROs (eg, a patient with a breached pain score received a link to the physical well-being page). Information resources, interactive resources (eg, videos, podcasts, self-help programs), and resources to facilitate effective communication with the general practitioner were provided. Patients with no breached ePROs received the “maintaining well-being” link. Resources were maintained on a public website, external to the health firewalls, and no identifiable information was included in the email.

### Selection of Controls

The “usual care” control group included all other patients receiving active treatment or follow-up care at participating centers during the study period. For comparability between the intervention and control samples, clinicians prescreened control patients as part of trial recruitment procedures; patients who did not meet the study eligibility criteria were excluded from the usual care group. Usual care consisted of standard clinical oncology practice, monitoring and managing patient issues, and management and OIS documentation of patient symptoms and issues as needed during clinical appointments.

### Outcomes

The primary outcome was ED presentations. Since patients could present at any hospital in their local health district, ED data was extracted from all hospitals with ED departments (n=8) in the two local health districts, during the trial period. A presentation to ED may or may not have required an admission. Secondary outcomes were (1) total time receiving chemotherapy during the study period and (2) referral to in-hospital allied health services (eg, psychology, social work, nutrition and dietetics, occupational therapy, physiotherapy). Primary and secondary outcome data were extracted from patients’ OISs.

Receipt of clinical alerts was automatically logged via the PROMPT-Care system email monitoring and OIS records of clinician notes. Responses to email alerts were recorded in the OIS and research staff recorded clinical actions (eg, referrals, information provision, issue already being managed). The number of clinical feedback reports opened in response to patient assessment completion was tabulated based on medical record logs.

PROMPT-Care compliance was monitored by calculating the proportion of patients completing assessments at expected time points within the first 6 months. Patient views of self-management domain pages, monitored using Google Analytics, were summarized as counts and proportion of resources.

At enrolment, patients completed a demographics survey (sex, marital status, education, employment, and language spoken at home); clinical (cancer site and stage) and treatment (chemotherapy, radiotherapy treatments, active treatment, and follow-up care) details were extracted from the OIS. Socioeconomic status was determined from the Index of Relative Socio-economic Disadvantage [19], a continuous score split into quintiles (1=most disadvantaged to 5=least disadvantaged).

### Sample Size

Propensity score matching was used to match control patients with intervention patients in a 4:1 ratio with regard to patient age, sex, and treatment status [20]. The study was powered to detect a minimum 14% between-group difference in the primary outcome—ED presentations. Assuming a 4:1 allocation to the control group versus intervention group and based on an assumed 1.4 ED presentations per patient during the study period for the control group [21], a minimum sample of 1760 patients (intervention: n=352; control: n=1408) was required to achieve 80% power and a two-sided statistical significance of 0.05.

### Statistical Analyses

Demographic and clinical characteristics were described as frequencies, mean scores, and percentages, with between-group comparisons using Chi-square tests or *t* tests. Intention-to-treat analyses were conducted in line with a prespecified analysis plan [12]. Multivariable negative binomial regression was used to identify between-group differences in ED presentation rates. To account for between-group differences in distribution of demographic and disease characteristics, we adjusted for disease stage, socioeconomic disadvantage, hospital site, and waiting time (time from diagnosis to start of PROMPT-Care). Allied health referrals were analyzed similarly. Multivariable Cox proportional hazards model was used to analyze length of time from start to end of chemotherapy, adjusting for stage of disease, treatment status, and socioeconomic disadvantage. A delayed entry model was used to specify how many days after the start of chemotherapy a patient started PROMPT-Care.

### Data Sharing

De-identified data will be available on request after all primary and secondary endpoints have been analyzed and published, and after signing of an agreement with the PROMPT-Care program. Requests for data sharing can be made to the corresponding author, including a research proposal that must be approved by the chief investigator team.

## Results

### Study Population

**Figure 3:**
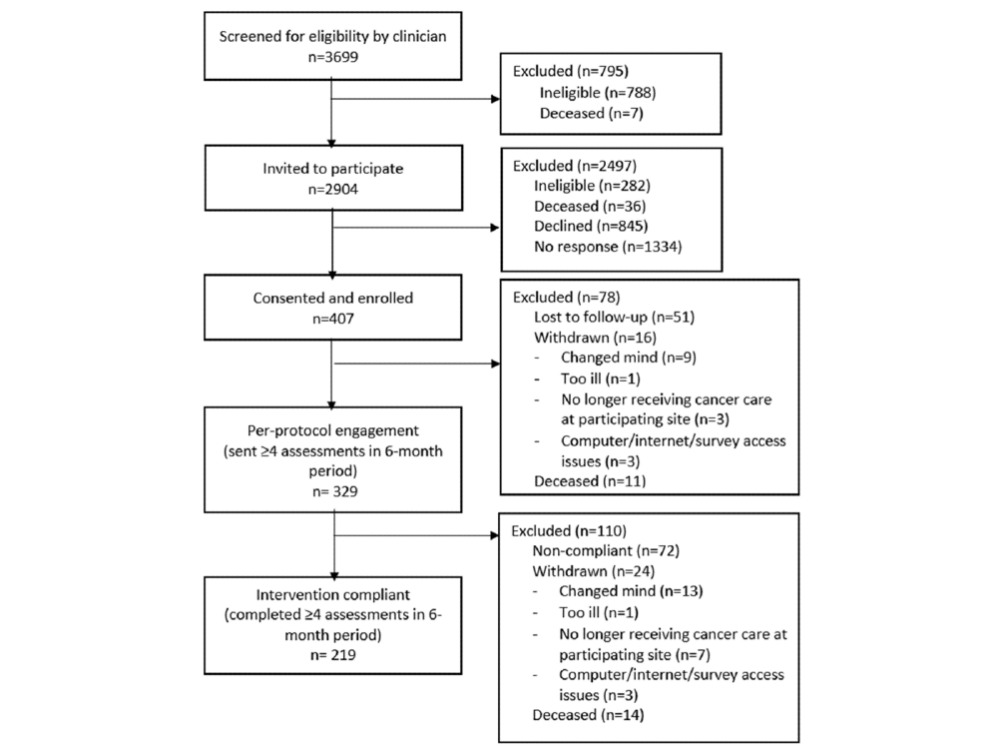
CONSORT diagram.

Between April 2016 and April 2018, clinicians prescreened 3699 patients against clinical and language exclusion criteria and invited 2904 (79%) to participate (Figure 3). A further 283 patients were ineligible, 36 were deceased, 845 declined, and 1334 did not respond. The remaining 406 patients were enrolled to the intervention group, with 328 (81%) receiving the “per-protocol intervention” (sent >4 assessments within 6 months postenrolment), due to administrative staff error sending assessment emails.

### Propensity Score Matching

Propensity score matching was used to match age, sex, and treatment status. Prior to matching, there were no significant differences in age and sex between the treatment groups. However, there was a higher proportion of control patients on active treatment (1157/1911, 60.5%) compared with patients in the intervention group (139/328, 42.4%) (*P*<.001). After matching there were no differences in age, sex, and treatment groups (Table 1).

After matching, the study groups were not significantly different at baseline in the site of cancer and waiting time, but the control group included significantly more patients from the most socioeconomically disadvantaged quintile and at clinical stage IV (*P*=.01; Table 1).

**Table 1:** Participant characteristics for intervention, control, and control participants matched for age, sex, and treatment status.

			PROMPT-Care^a^ (n=328)		Control (n=1911)		*P* value		Matched control (n=1312)		*P* value
Age (years), mean (range)			62.4 (25-86)		62.7 (18-96)		.61		62.3		.90
**Sex, n (%)**							.33				.45
	Male	133 (40.6)		720 (37.7)				502 (38.3)			
	Female	195 (59.5)		1191 (62.3)				810 (61.7)			
**Site of cancer, n (%)**							.50				.18
	Breast	132 (40.2)		854 (44.7)				620 (47.3)			
	Prostate	51 (15.6)		295 (15.4)				199 (15.2)			
	Colorectal	37 (11.3)		186 (9.7)				127 (9.7)			
	Respiratory	29 (8.8)		127 (6.7)				83 (6.3)			
	Gynaecological	16 (4.9)		83 (4.3)				43 (3.3)			
	Upper gastrointestinal	15 (4.6)		78 (4.1)				51 (3.9)			
	Skin	11 (3.4)		83 (4.8)				48 (3.7)			
	Oral	10 (3.1)		34 (1.8)				21 (1.6)			
	Other	27 (8.2)		171 (9)				120 (9.2)			
**Stage of disease^b^, n (%)**							.20				.01
	0/I	66 (22.1)		415 (21.7)				303 (23.1)			
	II	90 (27.4)		569 (29.8)				412 (31.4)			
	III	57 (17.4)		366 (19.2)				248 (18.9)			
	IV	80 (24.4)		357 (18.7)				209 (15.9)			
	Missing	35 (10.7)		204 (10.7)				140 (10.7)			
**Treatment received, n (%)**											
	Chemotherapy	105 (32)		561 (29.4)		.33		269 (20.5)		<.001	
	Radiotherapy	66 (20.1)		897 (46.9)		<.001		430 (32.8)		<.001	
**Treatment status, n (%)**							<.001				>.99
	Active treatment^c^	139 (42.46)		1157 (60.5)				558 (42.5)			
	Follow-up care	189 (57.6)		754 (39.5)				754 (57.5)			
**Socioeconomic status (IRSD)^d^, n (%)**							<.001				<.001
	1	54 (16.45)		609 (31.9)				403 (30.7)			
	2	98 (29.9)		490 (25.6)				340 (25.9)			
	3	52 (15.9)		275 (14.4)				207 (15.8)			
	4	35 (10.7)		197 (10.3)				124 (9.5)			
	5	90 (27.1)		340 (17.8)				238 (18.1)			
**Relationship status^b,e^, n (%)**							—^f^				—
	Single	71 (23.1)		—				—			
	Partnered	236 (76.9)		—				—			
**Education status^b,e^, n (%)**							—				—
	High school or less	122 (39.7)		—				—			
	Post-secondary education	185 (60.3)		—				—			
**Employment^c,e^, n (%)**							—				
	Employed	129 (42)		—				—			
	Retired	155 (50.5)		—				—			
	Other	23 (7.5)		—				—			
**Hospital site, n (%)**							—				<.001
	1	146 (44.5)		628 (32.9)				447 (34.1)			
	2	58 (17.7)		286 (15)				229 (17.5)			
	3	88 (26.8)		867 (45.4)				550 (41.9)			
	4	36 (11)		130 (6.8)				86 (6.6)			
Waiting time^g^, mean (range)			726.5 (1-5855)		662.2 (0-7458)		.22		785.1 (0-7458)		.31

^a^PROMPT-Care: Patient Reported Outcome Measures for Personalized Treatment and Care.

^b^Some level of missing data.

^c^Chemotherapy, radiotherapy, or both.

^d^IRSD: Index of relative socioeconomic disadvantage. 1=most disadvantaged; 5=least disadvantaged.

^e^Data extracted from the patient survey and are hence not available for the control group.

^f^Not available.

^g^Diagnosis date to PROMPT-Care start.

### Emergency Department Presentations

There were 314 ED visits from the 328 patients in the intervention group (0.96 ED visits per patient) and 1874 ED visits from the 1312 patients in the matched controls (1.4 ED visits per patient). After accounting for patient time in PROMPT-Care (intervention: 192,859 days; control: 1,006,956 days), the rates of ED visits were 16.2 per 100,000 patient days in the intervention group and 18.6 per 100,000 patient days in the matched controls.

After adjustment for stage, socioeconomic disadvantage, recruitment site, and waiting time in the multivariable negative binomial regression model with an offset of time in PROMPT-Care (to account for maldistribution of these variables between the groups), ED visits were significantly lower by 33% (*P*=.02) in the intervention group compared with the matched controls (Table 2).

**Table 2:** Comparison of emergency department presentations using negative binomial regression. Patients matched for age, sex, and treatment status.

		Univariate								Multivariable						
		RR^a^		Lower 95% CI		Upper 95% CI		*P* value		RR^a^		Lower 95% CI	Upper 95% CI		*P* value	
**Group**																
	Intervention	0.81		0.64		1.04		.10		0.75		0.60	0.95		.02	
	Control	Reference		—^b^		—		—		Reference		—	—		—	
**Stage**								<.001							<.001	
	0/I	Reference		—		—		—		Reference		—	—		—	
	II	1.50		1.16		1.96		.002		1.50		1.15	1.95		.002	
	III	2.93		2.20		3.91		<.001		2.56		1.92	3.43		<.001	
	IV	5.80		4.35		7.74		<.001		5.56		4.15	7.45		<.001	
	Missing	3.59		2.57		5.02		<.001		3.64		2.62	5.07		<.001	
**Socioeconomic status (IRSD)^c^**								.02							.003	
	1	1.81		1.25		2.61		.002		1.90		1.34	2.70		<.001	
	2	1.82		1.25		2.64		.002		1.38		0.96	1.98		.08	
	3	1.48		0.99		2.22		.06		1.77		1.21	2.59		.004	
	4	Reference		—		—		—		Reference		—	—		—	
	5	1.52		1.04		2.24		.03		1.40		0.96	2.03		.08	
**Recruitment site**								<.001							<.001	
	1	0.61		0.42		0.89		.01		0.91		0.63	1.32		.63	
	2	0.40		0.26		0.61		<.001		0.47		0.31	0.71		<.001	
	3	0.44		0.30		0.64		<.001		0.56		0.38	0.81		.002	
	4	Reference		—		—		—		Reference		—	—		—	
Waiting time			0.9998		0.9997		1.00		<.001		0.9999	0.9998		1.0000		.018

^a^RR: Relative risk.

^b^Not available.

^c^IRSD: Index of relative socioeconomic disadvantage. 1=most disadvantaged; 5=least disadvantaged.

### Time on Chemotherapy Treatment and Allied Health Referrals

Time on chemotherapy did not differ between the intervention and control groups (*hazard ratio*=0.96; *P*=.71; see Multimedia Appendix 1) after adjustment for stage, socioeconomic disadvantage, and recruitment site.

Allied health referrals were also not significantly different between intervention and control groups (*relative risk*=0.74; *P=*.20) after adjusting for stage, socioeconomic disadvantage, recruitment site, and waiting time (Table 3).

**Table 3:** Comparison of Allied Health referrals using negative binomial regression. Patients matched for age, sex, and treatment status.

		Univariate					Multivariable							
		RR^a^	Lower 95% CI	Upper 95% CI		*P* value			RR^a^		Lower 95% CI	Upper 95% CI		*P* value
**Group**														
	Intervention	0.44	0.27	0.72		<.001			0.74		0.48	1.16		.20
	Control	Reference	—	—		—			Reference		—	—		—
**Stage**					<.001								<.001	
	0/I	Reference	—	—		—			Reference		—	—		—
	II	2.73	1.62	4.61		<.001			2.16		1.30	3.60		.003
	III	7.29	4.14	12.86		<.001			6.10		3.52	10.56		<.001
	IV	10.61	5.96	18.89		<.001			9.62		5.51	16.79		<.001
	Missing	17.00	8.86	32.62		<.001			9.69		5.20	18.03		<.001
**Socioeconomic status (IRSD)^b^**					<.001								.14	
	1	3.61	1.79	7.29		<.001			2.22		1.15	4.31		.02
	2	2.26	1.11	4.58		.02			2.34		1.18	4.62		.01
	3	1.12	0.51	2.42		.78			1.59		0.75	3.41		.23
	4	Reference	—	—		—			Reference		—	—		—
	5	1.85	0.88	3.87		.10			2.12		1.05	4.27		.04
**Recruitment site**						<.001								<.001
	1	1.92	0.89	4.16		.10			2.58		1.23	5.41		.01
	2	0.14	0.05	0.36		<.001			0.21		0.08	0.54		.001
	3	3.52	1.63	7.57		.001			3.38		1.60	7.16		.001
	4	Reference							Reference					
Waiting time		0.9992	0.9990	0.9994		<.001			0.9993		0.9991	0.9995		<.001

^a^RR: Relative risk.

^b^IRSD: Index of relative socioeconomic disadvantage. 1=most disadvantaged; 5=least disadvantaged.

### System Utility

Patients (n=328) completed 2746 PROMPT-Care assessments. At baseline, the most common PRO (above threshold) were fatigue (128/328, 39%), tiredness (126/328, 38.4%), worry (108/328, 32.9%), general well-being (108/328, 32.9%), and sleep (79/328, 24.1%), aligning with the most frequently accessed self-management domain pages of physical well-being (680/1867, 36%) and emotional well-being (429/1867, 23%). The majority of patients (218/328, 66.4%) used the system as intended, completing four or more assessments within the 6-month intervention period.

Overall, 32% (893/2751) of clinical feedback reports were reviewed, the vast majority (729/893, 82%) by nursing staff and 17% by oncologists (149/893).

In total, 71% (233/328) of intervention patients generated a clinical alert. A total of 877 clinical email alerts were generated, with a mean of 31 (range 2-78) alerts per month during the 30-month study. Overall, 44% (383/877) of clinical alerts were reviewed by designated nurse care coordinators, resulting in 496 actions: in-clinic or telephone patient follow-up (302/496, 61%), no further follow-up deemed necessary (83/496, 17%), and telephone contact attempts but patient could not be reached (111/496, 22%). Issues were largely resolved through discussion (129/302, 43%) or information provision (98/302, 32%), with some health care professional referrals (75/302, 25%). 

## Discussion

### Principal Findings

We investigated PROMPT-Care implementation into routine clinical practice among diverse populations of patients with cancer. For adult patients with cancer, receiving active treatment or in follow-up care, algorithm-supported web-based systems utilizing PROs in routine practice resulted in fewer ED presentations. Prespecified secondary analyses showed no statistically significant associations in allied health referrals or time on chemotherapy and were likely underpowered to detect any change. Another factor to consider is the multimodal nature of the intervention where, in addition to clinical follow-up for above-threshold PROs, patients also received targeted resources enabling patients to self-manage minor issues where clinically appropriate. Further research should explore multimodal interventions such as PROMPT-Care that combine ePRO clinical implementation and appropriate patient self-management, as patients are responsible for managing their own care between hospital clinic visits.

The finding of reduced ED presentations observed in our study is also consistent with other web-based PRO studies. Basch et al [3,6] demonstrated that web-based symptom reporting with automated email alerts resulted in fewer ED visits. However, this was a single-site study, in a population with advanced disease receiving chemotherapy, with ED visits as a secondary outcome. Barbera et al [5] demonstrated that ESAS screening was associated with decreased ED visits, among patients with breast cancer; and Howell et al [22] showed reduced ED visits following ESAS screening in a prepost comparative cohort study. Additionally, to our knowledge, our study is the first to explore the impact of a multimodal intervention combining electronic PRO screening with clinical intervention and patient self-management.

### Integration and Clinical Use of ePRO Systems Into Routine Practice

In this pragmatic study, PROMPT-Care was implemented using available resources and workflows, with two important findings influencing future adoption. First, the automated clinical alerts prompted the majority of clinical report reviews, highlighting the importance of embedded triggers for reviewing above-threshold reports, which are otherwise passively accessible in the OIS. Second, nursing staff were instrumental in reviewing, triaging, and directly managing responses to clinical alerts, echoing many studies of nurse-led telephone navigator models of cancer care [23]. Additionally, with the relatively low number of clinical alerts generated each month, the automated alerts fit within existing workflows without creating onerous amounts of additional work. These findings will contribute to the evidence-based development and integration of ePRO systems into future models of routine care, to not only reduce the high demand on health services but also provide targeted systematic care to patients most in need.

### Strengths and Limitations

Our study addressed existing evidence gaps. We examined the impact on health service outcomes of an ePRO system implemented in routine practice settings, rather than a controlled research environment. We included a broad population of patients with cancer, enhancing generalizability. We monitored physical symptoms and psychosocial well-being and provided real-time feedback to care providers and patients. Intervention delivery to a broad cross-section of patients, across four centers providing comprehensive chemotherapy and radiotherapy treatment services, further enhances the study’s external validity. Hence, our findings are potentially generalizable to other clinical settings in countries with similar health systems, highlighting the importance of informing ePRO system implementation more broadly.

Our study also has some limitations. In our study, the intervention did not reach 100% of patients. First, response rate was low, possibly contributing to recruitment bias in treatment status and socioeconomic disadvantage. Second, patients unable to complete assessments in English or without access to a device and internet outside the hospital were excluded. While we acknowledge this limitation, our pilot study patients wanted remote electronic access to assessments [10], making this a critical component of our intervention design. Future interventions utilizing online or remote ePRO completion will likely be more accessible, with 86% of Australian households having internet access at home [24]. Third, due to administrative problems, only 81% of patients received the intervention per-protocol (sent ≥4 assessments in the first 6 months postrecruitment). Finally, there was limited follow-up (one email reminder) if monthly assessments were not completed, in contrast to nurses following up patients in the Basch et al [3] trial to ensure high adherence. Despite this, 67% of patients engaged with the system as intended, suggesting high intervention acceptability.

Clinically integrating PROs is challenging, even in centers with screening implemented for many years. Cancer Care Ontario’s systematic ESAS distress screening commenced in 2007, with rates increasing steadily from approximately 20% in 2009 to 59% in 2015, but remaining below the provincial target of 70% screened (range 31% at lowest performing to 91% at highest performing centers). Chow et al [25] also found that patients completed a brief distress screener 75% of the time they received a text message, suggesting feasibility of remote ePRO screening, as per our PROMPT-Care model.

Our assessment and alert frequencies are other limitations. Monthly assessments were selected to accommodate the longest response timeframe for the selected scales (Supportive Care Needs Survey-Screening Tool 9 “in the past month...”), and inclusion of follow-up as well as on-treatment patients. The Clinical Advisory Group decided that clinical alerts should be generated following two consecutive breaches [17] to minimize false-positive alerts, since patients on treatment were in regular contact with the cancer service; hence, any additional concerning issues would be readily identified between assessments. However, increasing assessment frequency may identify more acute symptoms (eg, pain), which likely result in ED presentations. Finding the balance between screening burden and timely alerts is an ongoing challenge.

Retrospective interrogation of systematically collected data shows that with systematic clinical implementation of distress screening, distress levels significantly predicted service utilization and referral rates [26], particularly to social and psycho-oncology services [27]. A key component of our effectiveness evaluation was to observe nurse uptake into workflows as part of routine practice with minimal intervention. This likely resulted in the low observed opening rates of clinical alerts. Further research is needed to explore implementation strategies that would encourage and support clinical staff to embed ePRO review and action into routine workflows.

### Future Research

This study enhances our understanding of how PROs inform cancer care and patient self-management beyond a randomized controlled trial and raises priority research questions. We know very little about reaching underserved patient populations. In particular, the extent to which ePRO systems like PROMPT-Care are acceptable and feasible for (1) assessing PROs in languages other than English and (2) informing patient-centered care in non-English subgroups is unknown. Furthermore, given the dearth of non-English resources to support patient self-management, applying cultural adaptation principles rather than simply translating existing resources into other languages [28] is a focus for future development.

### Conclusions

Although most previous research has evaluated ePRO systems with patients receiving adjuvant treatment, there is a compelling argument for eHealth systems like PROMPT-Care informing the care of the growing population of cancer survivors. Completing ePROs routinely can efficiently identify follow-up patients managing well, who can be supported with self-management resources rather than attending specialist follow-up appointments. ePROs can also detect issues of concern when patients do not have a scheduled appointment, prompting timely clinical care if required to avoid escalation in severity of issues. Research into the acceptability and cost-effectiveness of this model of care is required, but our research supports its acceptability and feasibility to patients and oncology staff. Research to date has predominantly focused on testing ePRO intervention efficacy. We have purposefully undertaken a pragmatic trial to better understand the effectiveness of ePRO systems in real-world settings, demonstrating that ePROs are likely to be adopted in routine care when integrated into the patient OIS and existing clinical workflows, allowing easy access by the care team. However, significant barriers exist for many cancer centers to do this. It is imperative that future research explore implementation questions, focusing on evaluating the processes and outcomes of ePRO systems adopted as business-as-usual.

## Appendix

Multimedia Appendix 1 Supplementary Table S1 - Time to end of chemotherapy.

## Abbreviations

ED: emergency department
ESAS: Edmonton Symptom Assessment Scale
HL7: Health Level Seven
OIS: oncology information system
PRO: patient-reported outcomes
PROMPT-Care: Patient Reported Outcome Measures for Personalized Treatment and Care
