# Supplementary material for: Web-Based Patient-Reported Outcome Measures for Personalized Treatment and Care (PROMPT-Care): Multicenter Pragmatic Nonrandomized Trial
Source: J Med Internet Res. 2020 Oct 29;22(10):e19685. doi: 10.2196/19685 (PMC7661255; doi:10.2196/19685)
Supplement: Multimedia Appendix 1 [file jmir_v22i10e19685_app1.docx]

|  | Univariate | | | | Multivariable | | | |
| --- | --- | --- | --- | --- | --- | --- | --- | --- |
|  | HR^a^ | Lower 95% CI | Upper 95% CI | P-value | HR | Lower 95% CI | Upper 95% CI | P-value |
| **Treatment Group** |  |  |  |  |  |  |  |  |
| Intervention | 1.01 | 0.84 | 1.22 | 0.9039 | 0.96 | 0.79 | 1.17 | 0.7103 |
| Control | Reference |  |  |  | Reference |  |  |  |
| **Stage** |  |  |  | 0.081 |  |  |  | 0.031 |
| 0/I | Reference |  |  |  | Reference |  |  |  |
| II | 1.01 | 0.67 | 1.52 | 0.9569 | 1.05 | 0.70 | 1.57 | 0.8045 |
| III | 0.88 | 0.59 | 1.32 | 0.5459 | 0.88 | 0.59 | 1.31 | 0.5269 |
| IV | 0.82 | 0.55 | 1.21 | 0.3155 | 0.78 | 0.53 | 1.17 | 0.2356 |
| Missing | 0.71 | 0.46 | 1.09 | 0.1132 | 0.73 | 0.48 | 1.10 | 0.1341 |
| **IRSD^a^** |  |  |  | 0.3455 |  |  |  | 0.4057 |
| 1 | 1.09 | 0.82 | 1.44 | 0.5735 | 1.05 | 0.80 | 1.38 | 0.714 |
| 2 | 1.08 | 0.81 | 1.45 | 0.5929 | 1.04 | 0.78 | 1.38 | 0.7832 |
| 3 | 1.16 | 0.83 | 1.62 | 0.3834 | 1.06 | 0.77 | 1.45 | 0.7356 |
| 4 | Reference |  |  |  | Reference |  |  |  |
| 5 | 1.29 | 0.96 | 1.73 | 0.0962 | 1.27 | 0.93 | 1.72 | 0.1315 |
| **Site** |  |  |  | 0.0885 |  |  |  | 0.0723 |
| 1 | 0.91 | 0.65 | 1.26 | 0.5622 | 0.80 | 0.57 | 1.14 | 0.2202 |
| 2 | 1.16 | 0.77 | 1.73 | 0.482 | 1.12 | 0.75 | 1.69 | 0.5764 |
| 3 | 0.82 | 0.58 | 1.14 | 0.2383 | 0.78 | 0.55 | 1.10 | 0.1516 |
| 4 | Reference |  |  |  | Reference |  |  |  |

^a^HR: Hazard Ratio

^b^IRSD: Index of relative socioeconomic disadvantage. 1=most disadvantaged; 5=least disadvantaged.
